# VHL-P138R and VHL-L163R Novel Variants: Mechanisms of VHL Pathogenicity Involving HIF-Dependent and HIF-Independent Actions

**DOI:** 10.3389/fendo.2022.854365

**Published:** 2022-03-21

**Authors:** Cecilia Mathó, María Celia Fernández, Jenner Bonanata, Xian-De Liu, Ayelen Martin, Ana Vieites, Gabriela Sansó, Marta Barontini, Eric Jonasch, E. Laura Coitiño, Patricia Alejandra Pennisi

**Affiliations:** ^1^ Centro de Investigaciones Endocrinológicas Dr. César Bergadá (CEDIE), Consejo Nacional de Investigaciones Científicas y Técnicas- Fundación de Endocrinología Infantil (CONICET-FEI) División de Endocrinología, Hospital de Niños Dr. Ricardo Gutiérrez, Buenos Aires, Argentina; ^2^ Laboratorio de Química Teórica y Computacional (LQTC), Instituto de Química Biológica, Facultad de Ciencias and Centro de Investigaciones Biomédicas (CEINBIO), Universidad de la República, Montevideo, Uruguay; ^3^ University of Texas MD Anderson Cancer Center, Houston, TX, United States

**Keywords:** VHL, von Hippel–Lindau, novel variants, P138R, L163R, functional characterization, molecular dynamics, simulations

## Abstract

The von Hippel–Lindau (VHL) disease is an autosomal dominant cancer syndrome caused by mutations in the *VHL* tumor suppressor gene. VHL protein (pVHL) forms a complex (VBC) with Elongins B-C, Cullin2, and Rbx1. Although other functions have been discovered, the most described function of pVHL is to recognize and target hypoxia-inducible factor (HIF) for degradation. This work comprises the functional characterization of two novel variants of the VHL gene (P138R and L163R) that have been described in our center in patients with VHL disease by *in vitro*, *in vivo*, and *in silico* approaches. *In vitro*, we found that these variants have a significantly shorter half-life compared to wild-type VHL but still form a functional VBC complex. Altered fibronectin deposition was evidenced for both variants using immunofluorescence. *In vivo* studies revealed that both variants failed to suppress tumor growth. By means of molecular dynamics simulations, we inspected *in silico* the nature of the changes introduced by each variant in the VBC complex. We have demonstrated the pathogenicity of P138R and L163R novel variants, involving HIF-dependent and HIF-independent mechanisms. These results provide the basis for future studies regarding the impact of structural alterations on posttranslational modifications that drive pVHL’s fate and functions.

## 1 Introduction

The von Hippel–Lindau (VHL) disease is a hereditary autosomal dominant syndrome (1, 2) that predisposes to the formation of cysts and benign and malignant tumors in different organs (3). Clinically, VHL disease can be divided into two subtypes based on the absence (type 1) or presence (type 2) of pheochromocytoma (4).

VHL disease’s incidence ranges from 1/36,000 to 1/45,000 live births (3, 5) and is caused by mutations in the *VHL* tumor suppressor gene, which is located in the short arm of chromosome 3 (3p25-26) (3). Its coding sequence spans three exons and encodes a 213-amino acid protein (pVHL) widely expressed in human tissues (4, 6).

The correct folding of pVHL is coupled to the formation of the VBC complex with Elongin B and Elongin C (7, 8). The VBC complex together with Cullin 2 is part of the substrate-binding subunit of an E3 ubiquitin ligase that negatively regulates the expression of the hypoxia-inducible factors (HIFs) (9, 10). At normal oxygen level, HIF-α is hydroxylated at proline residues, in this form is recognized by pVHL, leading to rapid ubiquitination and degradation by the proteasome (11, 12). In hypoxic conditions, the prolyl-hydroxylases are inactive and HIF-α is stabilized, dimerizes with HIF-β (constitutively expressed), and translocates to the nucleus (12, 13). The dimer functions as a transcription factor, negatively regulating the expression of diverse hypoxia-inducible genes involved in metabolism, angiogenesis, and apoptosis (12, 14). In the past years, research has demonstrated that the SUMOylation of pVHL by the protein RSUME prevents the formation of the VBC complex, thus HIF-α is not degraded even under normal oxygen conditions (15, 16). On the other hand, pVHL has HIF-independent actions, such as microtubule stabilization (17), primary cilium formation (18), and extracellular matrix fibronectin assembly (19, 20), which are also important for tumor development.

To this day, more than 500 *VHL* mutations have been reported according to the Human Gene Mutation Database (HGMD^®^ Professional 2020.3, accessed on November 5, 2020). Interestingly, most of the families presenting with pheochromocytoma (type 2 VHL disease) harbor missense mutations, while families with type 1 VHL disease usually present with gene deletions or nonsense mutations (21–24). In the present work, we performed functional characterization of two genetic variants (P138R and L163R) that have been described at our center in patients with VHL disease (25). P138R variant was identified in 5 patients of a family with Type 2B VHL. L163R variant was identified in 2 patients of a family with pheochromocytoma only (Type 2C VHL). The P138R variant implies the change of a proline for an arginine in the β domain of pVHL, involved in the interaction with HIF-α, while the L163R (25) variant is located in the α domain, involved in the union with Elongins B and C. Through *in vitro*, *in vivo*, and *in silico* studies, we demonstrated the pathogenicity of P138R and L136R variants affecting not only pVHL capacity to form HIF’s recognition complex and its functioning in pseudo hypoxic conditions but also some of HIF’s independent actions.

## 2 Materials and Methods

### 2.1 Site-Directed Mutagenesis

The vector *VHL*-wild-type (WT)-Venus-Retro (26) and the Quikchange II XL Site-Directed Mutagenesis Kit were used following manufacturer’s protocols to perform the specific mutations P138R (CCA➔CGA) and L163R (CTC➔CGC). Mutations were verified by DNA sequencing in ABI PRISM 310 Genetic Analyzer (Applied Biosystems, Foster City, CA, USA).

### 2.2 Stable Cell Line Development

HEK293T cells were used as a helper cell line in order to obtain retrovirus with the desired vectors as previously described by Ding et al. (27). Briefly, HEK293T cells were transfected with 3 different vectors: 1) pcGp, 2) pVSVG, and 3) either one of the following: GFP-Retro/*VHL*-WT-Venus-Retro/*VHL*-P138R-Venus-Retro/*VHL*-L163R-Venus-Retro using Lipofectamine 3000 (Invitrogen, Carlsbad, CA, USA). Upon assembly, supernatant was used to infect RCC 786-0 cells (ATCC^®^ CRL-1932™, American Type Culture Collection, Manassas, VA, USA), and after 20h, selection was performed with 1 mg/ml of G418 antibiotic (Sigma Aldrich, St. Louis, MO, USA). Four different cell lines were obtained expressing green fluorescent protein (GFP), *VHL*-WT-Venus, VHL-P138R-Venus, and VHL-L163R-Venus. All cell lines were cultured in high-glucose Dulbecco’s modified Eagle’s medium (DMEM) supplemented with 10% fetal bovine serum (FBS) and maintained at 37°C in a humidified 5% CO_2_ environment.

### 2.3 Western Blotting

Proteins were obtained as previously described (28) and resolved on a 12.5% sodium dodecyl-sulfate polyacrylamide gel electrophoresis (SDS-PAGE). After transferring to polyvinylidene fluoride (PVDF) membranes, blots were blocked and probed with different primary antibodies: VHL (BD Biosciences, # 556347, diluted 1/5,000), GFP (Santa Cruz, sc-8334, diluted 1/1,000), HIF-2α (Novus Biologicals, NB100-122, diluted 1/1,000), β-actin (Cell Signaling, #4970, diluted 1/1,000), Elongin B (Santa Cruz, sc-133090, diluted 1/500), and Elongin C (Santa Cruz, sc-1559, diluted 1/500). The following secondary antibodies were used accordingly: anti-rabbit (Cell Signaling, #7074, diluted 1/5,000), anti-goat (Santa Cruz, sc 2020, diluted 1/2,000), and anti-mouse (Cell Signaling, #7076, diluted 1/2,000).

### 2.4 Cell Treatments

Cell lines were seeded on 6-well plates and incubated with 50 µg/ml cycloheximide to interfere with protein synthesis, or 5 µg/ml MG132 to inhibit the proteasome, or 100 µM CoCl_2_ (29) to simulate hypoxia. After treatment, proteins or RNA was extracted.

### 2.5 Immunoprecipitation

The amount of protein coming from GFP, WT VHL-Venus, P138R VHL-Venus, and L163R VHL-Venus cell lines was determined by Bradford assay, and 1 mg of protein was immunoprecipitated using GFP-Trap^®^_A kit (Chromotek GmbH, Germany). The immunocomplexes were detected by Western blot using the antibodies described above. Protein from WT VHL-Venus cell line was used as positive control and that from the cell line expressing GFP as a negative one.

### 2.6 Real-Time PCR

Total RNA from the different cell lines was extracted with Direct-Zol RNA Kit (Zymo Research, Irvine, CA, USA) following manufacturer’s protocol. To perform RT-qPCR, 1 µg of RNA from each sample was used together with random hexamers and Super Script II (Invitrogen, Carlsbad, CA, USA). Resulting cDNA was diluted by 1:10, and 3 μl from each dilution was subject to qPCR in triplicate using Kapa Syber Fast qPCR master mix (Kapa Biosystems, Boston, MA, USA) in Step One Plus Real-Time PCR System (Life Technologies, Carlsbad, CA, USA). mRNA values were calculated using relative quantitation method and are presented as fold change compared to control conditions. Specific primers were designed to assess fibronectin, vascular endothelial growth factor A (VEGF-A), and glucose transporter 1 (GLUT1) normalized to TATA box-binding protein (TBP) or *VHL* and α subunit α of HIF-2 (HIF-2α) normalized to glyceraldehyde 3-phosphate dehydrogenase (GAPDH).

### 2.7 Fibronectin Deposition by Immunofluorescence

Using anti-fibronectin antibody combined with a secondary antibody conjugated with Cy5, matrix deposition by all cell lines was analyzed according to the protocol of Debnath et al. (30). Briefly, cells were plated on coverslips, fixed, and permeabilized after 6 days of culture. Nuclei were dyed with Hoechst (5 µg/ml), and pictures were taken on a Carl-Zeiss AxioScope A1 microscope.

### 2.8 Xenografts

Immunodeficient mice [N:NIH (S)-Fox 1^nu^] were housed in standard conditions of 12-h light/12-h dark cycle with water and food *ad libitum*, in accordance with National Institutes of Health guide for the care and use of laboratory animals (31).

A solution of 1 × 10^7^ viable cells was injected subcutaneously on 6–8-week-old male mice and monitored weekly for tumor development. At 16 weeks post cell injection or when tumor reached 2-cm diameter, mice were sacrificed, and tumor histology was evaluated by hematoxylin and eosin (H&E) staining.

All animals were treated and cared for in accordance with standard international animal care protocols. All procedures were approved by the Animal Care and Use Committee of the Hospital de Niños Dr. Ricardo Gutiérrez.

### 2.9 Database Search and Online Predictions

We searched for these variants in the Genome Aggregation Database (gnomAD) (32), dbSNP (33), and ClinVar (34) databases to look at allele frequency, and if they had been reported by other groups. We also used online tools that predict the effect of protein variants: SIFT (35), Polyphen (36), Mutation Taster (37), and Human Splicing Finder (38). To classify these variants according to the American College of Medical Genetics Guidelines (39), we used VarSome (40).

### 2.10 *In Silico* Studies: Molecular Dynamics Simulations

The crystal structure of a human VBC: HIF-1α complex PDB 4AJY (X-Ray diffraction, 1.73 Å resolution) was used as starting structure (41). Missing residues of EloC (amino acids 106–118) were added using the SWISS-MODEL workspace (42, 43). The following six macromolecular systems were considered: WT and P138R and L163R variants of pVHL inserted in VBC: HIF-1α complexes, both under normoxia or hypoxia (the latter simulated replacing Hyp564 by Pro564 in HIF-1α). Lacking experimental structures of the two variants considered, *in silico* mutations were introduced by replacing the residue of interest at the native structure using the SWISS-PDB Viewer software (42). Protonation states of titratable residues were determined with PROPKA 3.0 (44), then all missing hydrogen atoms were added with the ProToss utility of the Proteins Plus server. All the systems were solvated with a truncated-octahedral box of TIP3P water 12 Å around the solute and neutralized with K^+^ ions using the *leap* module of AmberTools17 (45). Each of the systems was minimized (2,000 steps applying a 500 kcal mol^−1^ Å^−2^ harmonic potential over solute atoms, followed by 20,000 steps without restraints), then heated to 310 K [500 ps molecular dynamics (MD) simulation in NVT ensemble] and equilibrated at 1 atm (1 ns MD simulation at 310 K in NPT ensemble), prior to run 400 ns of productive MD simulations (NPT, 310 K and 1 atm). Minimizations and MD simulations were carried out with the *pmemd.cuda* module of AMBER16 (45). Protein residues were treated using the AMBER *ff14SB* force field. An integration step of 2 fs was used, constraining bonds involving hydrogen with SHAKE algorithm (46). Temperature and pressure were controlled applying the Langevin thermostat (47) and the Monte Carlo barostat (48), respectively. An 8.0-Å cutoff was used for direct non-bonded interactions, and the Particle Mesh Ewald (PME) method (49) was applied to long-range electrostatic interactions. Trajectory processing and analysis were performed with *cpptraj* module of AmberTools 17. Trajectory convergence was monitored following Cα-RMSDs, and flexibility was examined by means of per-residue Cα-RMSF. Snapshots of the trajectory were clustered into 5 clusters—each one with a representative structure—using a hierarchical agglomerative algorithm. Binding free energies of HIF-1α to the VBC complex were calculated using the MM-PB(GB)SA methods (50). For those calculations, the first 50 ns of the trajectories were discarded, then 100 snapshots separated by 3.5 ns were used. Representative structures of clusters with appreciable population (>10%) were used to calculate the electrostatic potential of VBC using the APBS software (51) implemented in the APBS/PDB2PQR web server (52).

### 2.11 Statistical Analysis

For real-time PCR analysis, one-way ANOVA was used with a Tukey test post evaluation. The chi-square test was used to analyze the differences in tumor incidence, and crosstabs were created. Statistical significance was defined as a p-value <0.05, and all data were graphed as mean ± standard deviation unless indicated otherwise.

## 3 Results

### 3.1 P138R and L163R pVHL Variants Exhibit Lower Protein Levels Than Wild-Type pVHL

**Figure 1:**
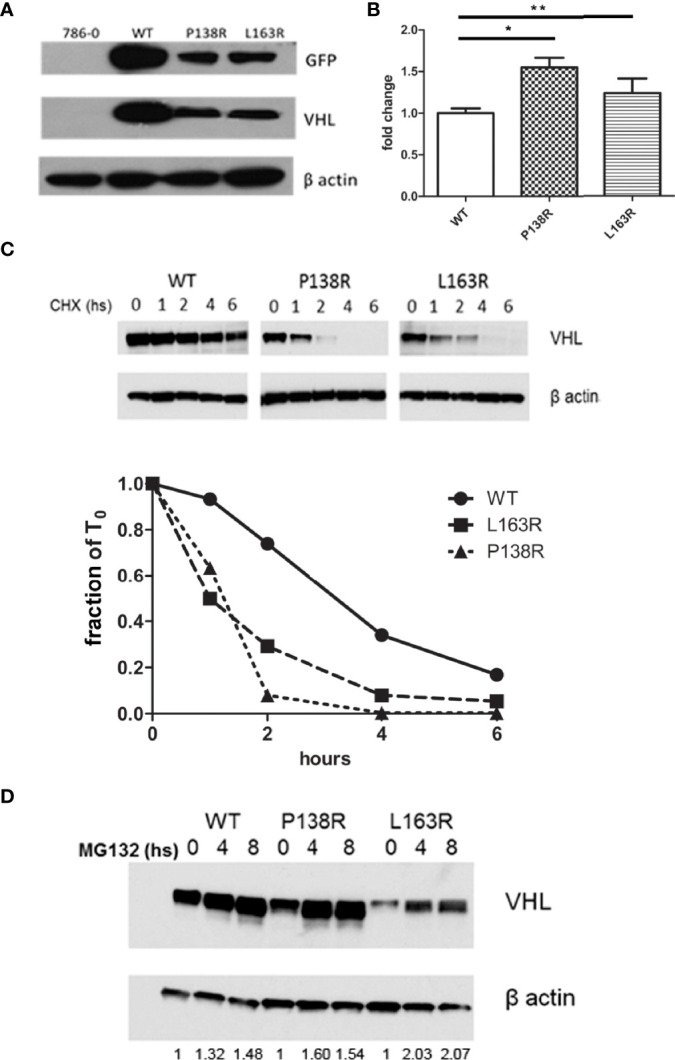
Reduction in protein levels and half-life for P138R and L163R pVHL variants. **(A)** Representative Western blot showing the levels of GFP and VHL protein obtained in each cell line and β-actin as loading control. **(B)** Expression of VHL measured by qrPCR and graphed as fold change for P138R and L163R pVHL variants compared to pVHL WT. *p < 0.0001, **p = 0.0424, one-way ANOVA and Tukey’s posttest. **(C)** Proteins levels obtained by Western blot after treatment with 50 µg/ml cycloheximide to inhibit protein translation. Quantification was done in order to plot the proportion of protein levels on the different time points evaluated. The dotted line indicates the 50%. **(D)** Inhibition of proteasome by 5 µg/ml MG 132 for cell lines expressing WT, and P138R and L163R pVHL variants. Results are shown by a representative Western blot for VHL and β-actin. Relative quantification of the bands is shown under each line.

We analyzed the effect of P138R and L163R novel variants on VHL protein stability using Venus-tagged proteins. Human 786–0 RCC cell line (*VHL*-deficient) was infected with retroviral vectors to stably express VHL-P138R-Venus, VHL-L163R-Venus, and *VHL*-WT-Venus. Protein levels for both variants were significantly lower than those for VHL-WT-Venus (**Figure 1A**). Assessed by RT-qPCR, mRNA levels showed that VHL-P138R-Venus and VHL-L163R-Venus variants were similar and even higher than VHL-WT-Venus mRNA levels (**Figure 1B**), suggesting that transcription levels are not responsible for the differences in protein levels evidenced by Western blot.

Cell lines were treated with cycloheximide to inhibit protein translation and enable the determination of half-lives for both VHL variants and WT pVHL. After 6 h, results showed that *VHL*-P138R-Venus and VHL-L163R-Venus have a significantly shorter half-life (≈1.2 h and 1 h, respectively) compared to that of VHL-WT-Venus (≈3.4 h) (**Figure 1C**).

Inhibiting the proteasome with MG132 (proteasome inhibitor) significantly increased both variants’ protein levels, achieving quantities comparable to WT pVHL levels after MG132 treatment for the case of P138R and slightly lower for L163R (**Figure 1D**).

### 3.2 VBC Complex Formation Is Apparently Diminished but Still Functional for P138R and L163R

**Figure 2:**
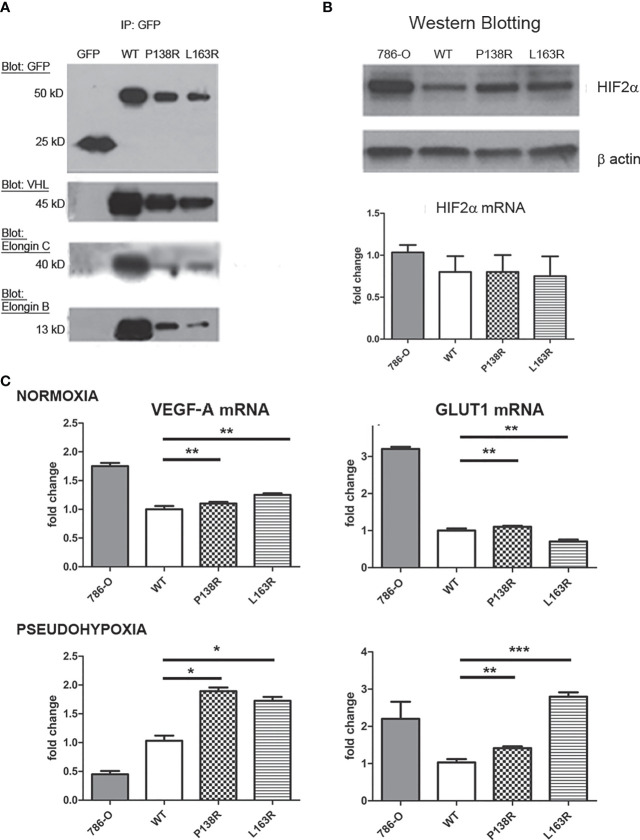
P138R and L163R pVHL variants form less VBC complexes without losing functionality. **(A)** Representative Western blot showing immunoprecipitation of GFP-trap for each cell line expressing GFP, VHL-WT, P138R, or L163R. Membranes were blotted with anti-GFP, anti-VHL, anti-Elongin C, and anti-Elongin B. **(B)** Representative Western blot showing the levels of HIF-2α protein and mRNA measured by RT-qPCR and graphed as fold change for 786-O, WT, P138R, and L163R cell lines. **(C)** *VEG-F* and *GLUT1* mRNA expression was calculated by RT-qPCR under normoxia or 24 h of pseudohypoxia generated with 100 µM CoCl_2_. Results are presented as fold change relative to pVHL WT expression. ns, not significant; *p < 0.0001, **p = 0.0401, ***p = 0.0002, one-way ANOVA and Tukey’s posttest.

To date, pVHL’s most described function is its interaction and consequent downregulation of HIF-α protein subunits (53). To this end, pVHL needs to form the VBC complex (pVHL-Elongin B-Elongin C). Immunoprecipitation of GFP Trap showed a specific band of 25 kD for GFP alone and 50 kD on cells expressing GFP-pVHL-Venus Tag (**Figure 2A**). Consistent with previous results (**Figure 1A**), pVHL levels are different for the WT and P138R and L163R variants, resulting in less coimmunoprecipitation of Elongin B and C for the variants compared to WT *VHL* cell line (**Figure 2A**). We calculated the ratio between the bands obtained: Elongin C/pVHL and Elongin B/pVHL for WT pVHL, P138R and L163R pVHL-expressing cell lines. Ratios were normalized to WT pVHL’s set as 1, and we observed that P138R immunoprecipitates less Elongin B and Elongin C (approximately 0.6) and L163R manages to immunoprecipitate a similar proportion of Elongin C but a lower quantity of Elongin B (0.25).

Since VBC complex was evidenced for both variants, we sought to evaluate its functionality. Firstly, the capacity of pVHL variants to downregulate HIF-2α was assessed. HIF-2α is overexpressed in the parental cell line used (786-0) (54), and its levels decrease significantly in the derived cell line expressing *VHL*-WT-Venus (**Figure 2B**, lanes 1 and 2). Protein levels for both P138R and L163R cell lines (**Figure 2B**, lanes 3 and 4) were intermediate for HIF-2α assessed by Western blot, although mRNA levels did not change in the different cell lines (**Figure 2B**). To evidence the consequence of these intermediate levels of HIF-2α protein, we quantified mRNA levels of two of its downstream targets: *VEGF-A* and *GLUT1* using qRT-PCR in normoxic and pseudohypoxic conditions (**Figure 2C**). Despite different HIF-2α protein levels, mRNA levels in normoxia for *VEGF-A* and *GLUT1* were similar among cell lines expressing WT and P138R and L163R pVHL (**Figure 2C**, upper panel). Under pseudohypoxic conditions, we found significantly higher levels of *VEGF-A* and *GLUT1* mRNAs on the variant cell lines compared to the one expressing WT pVHL (**Figure 2C**, lower panel).

### 3.3 Altered Fibronectin Deposition in P138R pVHL and L163R pVHL With Different RNA Levels

**Figure 3:**
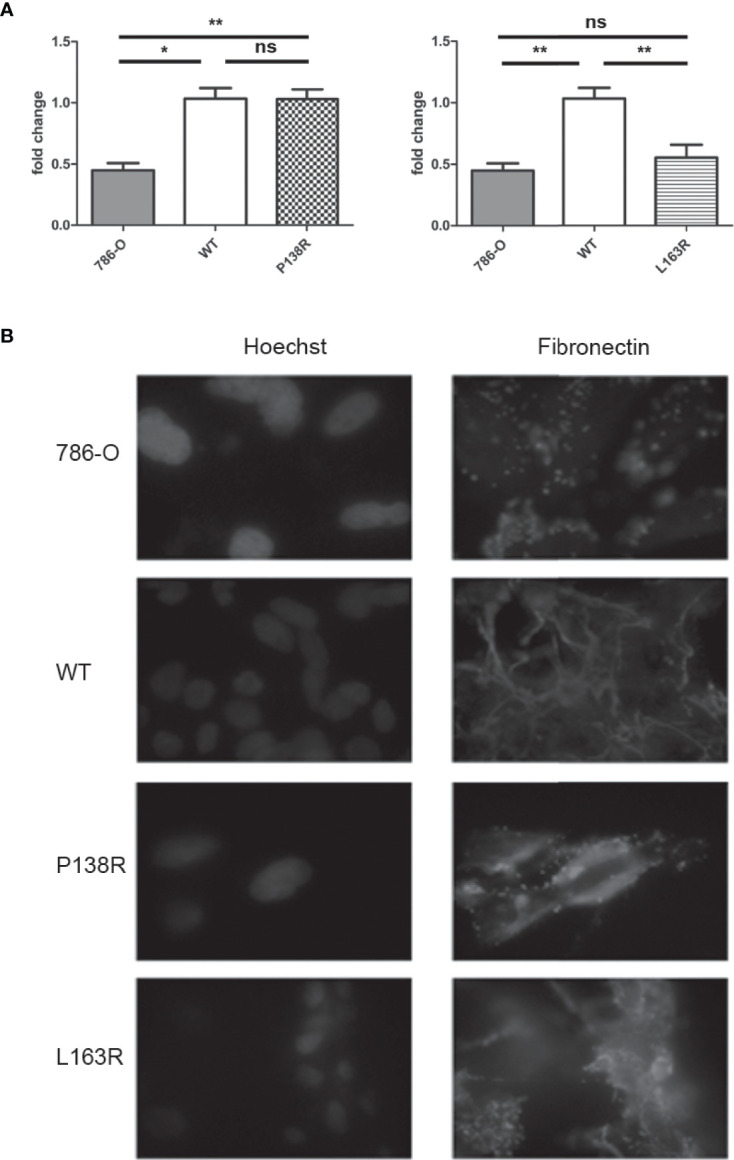
Differences in mRNA fibronectin expression for P138R and L163R pVHL variants with similar disrupted deposition patterns. **(A)** Fibronectin mRNA expression of 786-O, WT, P138R, and L163R cell lines. Results are presented as fold change compared to WT cells. Values are expressed as ± SD of three independent experiments performed in triplicate. ns, not significant; *p = 0.0011, **p = 0.0030, one-way ANOVA and Tukey’s posttest. **(B)** Cell lines were cultured on coverslips to assess fibronectin deposition with anti-fibronectin Cy5 conjugated (in red) by immunofluorescence. Nuclei were dyed with 5 μg/ml Hoechst as shown in blue. Images were taken at ×40 on a Carl-Zeiss AxioScope A1 microscope.

pVHL is known to regulate fibronectin mRNA levels, although the underlying molecular mechanism has not been yet described. We assessed fibronectin mRNA levels in the 786-0 and 786-0-derived cell lines expressing *VHL*-WT-Venus, VHL-P138R-Venus, and *VHL*-L163R-Venus by RT-qPCR. Cells expressing WT-VHL have higher fibronectin mRNA levels than the parental 786-0, which is pVHL null (**Figure 3A**). Regarding the variants, P138R expression shows similar fibronectin mRNA levels to that of WT-*VHL*-expressing cell line. On the other hand, L163R expression resulted in diminished fibronectin mRNA levels and significantly different to the WT-*VHL* but comparable to the levels obtained for 786-0 cell line (**Figure 3A**).

Fibronectin expression *per se* does not ensure its proper extracellular matrix organization. Using immunofluorescence, we evidenced fibronectin deposition in the 786-0 cell line as a dotted pattern, while in *VHL*-WT-Venus resulted in fibrillar network of fibronectin deposition (**Figure 3B**). Both variants, P138R and L163R, failed to generate this fibrillar organization, demonstrating a pattern similar to that observed in the parental 786-0 cell line where pVHL is absent (**Figure 3B**).

### 3.4 Cells Expressing P138R and L163R pVHL Do Not Suppress Tumor Growth as Wild-Type pVHL Does

**Figure 4:**
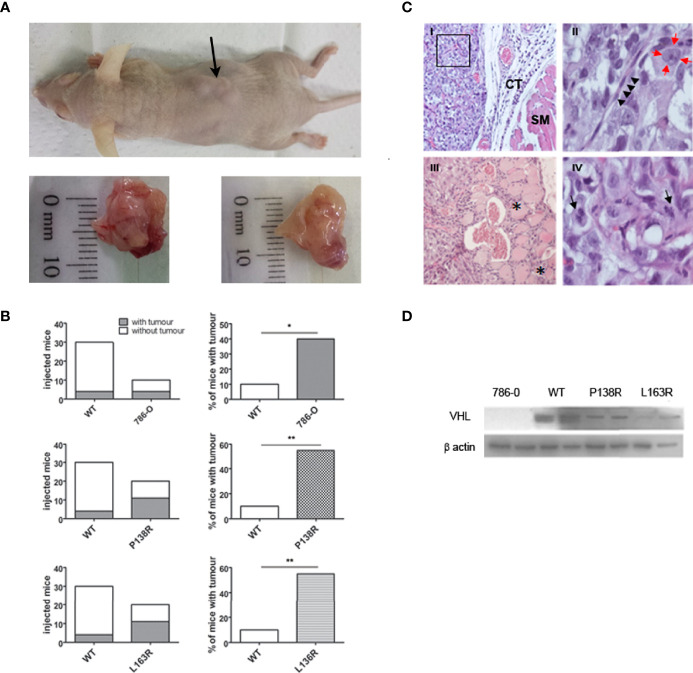
*In vivo* studies showed tumor development for P138R and L163R pVHL variants. **(A)** Representative picture of nude mice and the tumors developed. The arrow points toward a tumor (upper panel). The bottom panel shows the macroscopical aspect of the tumors. **(B)** Left plots represent the incidence obtained for each cell line when injected on immunodeficient mice, and percentages are plotted on the right panels. ns, not significant; *p = 0.0306, **p = 0.0005, two-tailed chi-square test. **(C)** Histological features of the experimentally obtained tumors and stained with H&E. Panel I, Tumor cells distributed as lobes of polyhedral cells separated by fine fibers of connective tissue (CT) and striated muscle (SM) ×20 (Panel I). Panel II, a magnification of a sector of panel I shows a connective septum with central endothelial nuclei corresponding to the capillary vessel (marked with black arrowheads), surrounded by tumor cells with nuclei (red arrows) with prominent central nucleolus; ×100. Panel III presented tumor infiltrating the neighboring striated muscle, and the asterisks (*) indicate traces of tumor progression between the muscle bundles. Panel IV shows mitotic figures indicated with black arrows; ×100. **(D)** Representative Western blot showing the expression of VHL protein in the tumors developed by 786-O, WT, P138R, and L163R cell lines. β-Actin was blotted as loading control.

To test the tumor suppressor role of the novel variants, we injected the cell lines expressing WT-*VHL* and P138R and L163R pVHL into male nude mice. Also, 786-0 cell line was injected as an internal control for the experiments. In our hands, visible tumors were developed, on average, 9 weeks after injection for all the tested cell lines (**Figure 4A**).

As expected, the ratio between the number of tumors developed and the number of sites injected was significantly higher in 786-0 compared to the cells expressing the WT-*VHL* protein. Moreover, P138R and L163R pVHL-expressing cells developed more tumors when compared to WT-*VHL* cell line (**Figure 4B**). Contingency tables were obtained, showing a significant difference between P138R, L163R, or 786-0 cells with WT pVHL, where tumors developed in 55% (11/20 for both variants) or 40% (4/10 for 786-0 cells) of the sites injected compared to a 10% for WT pVHL (3/30) (**Figure 4B**). Also, the variants showed a similar ratio of developed tumors to that of the parental cell line.

H&E staining confirmed that developed tumors had histological characteristics that are compatible with clear cell renal carcinoma (**Figure 4C**). These solid tumors were composed of atypical polyhedral cells that have a large, acidophilic, or optically empty cytoplasm with large nuclei where its membrane was observed thickened and a prominent central nucleolus. Cells are grouped into clusters separated by thin collagen tracts through which small blood vessels pass (**Figures 4C**, I, II). Tumors had infiltrating growth toward neighboring tissues (**Figures 4C**, III) and showed histological signs of proliferative activity, evidenced by the numerous mitotic figures found (**Figures 4C**, IV).

pVHL protein expression was verified on tumors developed by 786-0 cells, WT pVHL, P138R, and L163R cell lines by Western blot. As shown in **Figure 4D**, pVHL was not detectable on 786-0 cells and had higher levels on WT pVHL-expressing cells compared to both variants (P138R and L163R).

### 3.5 Database Search and Online Predictions

The results of our database and online prediction tools are summarized in **Table 1**.

**Table 1:** Databases and online predictions for our pVHL variants.

Variant	ACMG Classification using VarSome	Databases			Mutation Effect Predictions			
		gnomAD (v3.1.2&2.1.1)	dbSNP	ClinVar	SIFT	Polyphen	Mutation Taster	Human Splicing Finder
P138R	Likely pathogenic	NA	NA	NA	Affect protein function	Probably damaging	Deleterious	New donor splice site
L163R	Pathogenic	NA	rs28940297	VUS	Affect protein function	Probably damaging	Deleterious	No significant impact on splicing signals

NA, not available; VUS, Variant of Unknown Significance.

Our variants were not found in the Genome Aggregation Database (gnomAD) that includes thousands of genomes and exomes; this information allows us to infer that they have a very low allelic frequency. Most of the effect prediction tools used suggest that both variants are deleterious. L163R was previously reported by our group and reported in ClinVar by a genetic testing laboratory that classifies it as a variant of unknown significance (VUS). Using VarSome to follow the ACMG guidelines for classification of new variants, they are classified as likely pathogenic (P138R) and pathogenic (L163R).

### 3.6 *In Silico* Studies of VBC: HIF-1α Complexes by Molecular Dynamics Simulations

**Figure 5:**
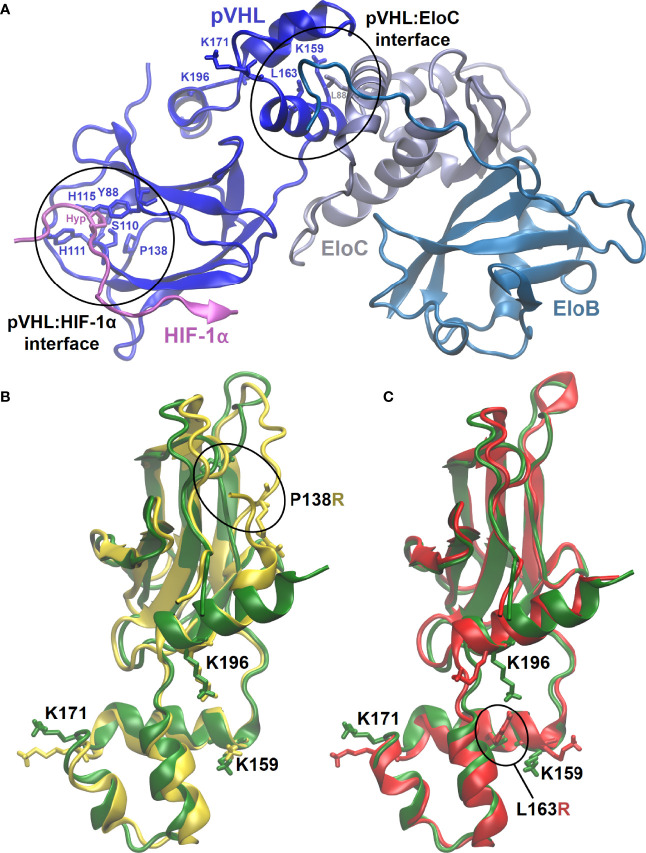
3D representative structures from MD simulations. **(A)** VBC complex with pVHL : HIF-1α and pVHL : EloC interfaces where variants are located circled and evidencing relevant residues. **(B, C)** Overlapped representative structures for the most populated clusters from 400-ns MD simulation under normoxia. Circled residues correspond to pVHL variants amino acids P138R and L163R in **(B, C)**, respectively. Color code: green, wild type pVHL; yellow, P138R pVHL variant; red, L163R pVHL variant.

MD simulations enabled us to inspect at a molecular level the effects of introducing P138R and L163R pVHL variants in the VBC: HIF complex structure (**Figure 5A**) and stability, flexibility of the protein components, and other features relevant toward molecular recognition of pVHL by HIF (here represented by a 559-577 peptide fragment from HIF-1α containing either hydroxyproline Hyp564 or P564 in a carboxyl-terminal oxygen-dependent CODD motif, as representative of normoxia and hypoxia, respectively) in the VBC complex and by other possible interactors (**Figure 5A**).

All of the six MD 400-ns simulations promptly converged, showing formation of structurally stable complexes in all the cases. Introducing variants P138R and L163R in pVHL (**Figure 5B**) appears not to considerably disrupt HIF-1α binding to VBC under normoxic conditions: as shown in **Table 2**, the three complexes display similar binding strength values. Although VBC: HIF-1α complexes still form as evidenced *in vitro* (**Figure 2A**), binding strength is significantly reduced in all the cases under hypoxia, particularly for variant P138R (**Table 2**).

**Table 2:** MMPB(GB)SA-binding free-energies (Δ_b_ *G*) for VBC: HIF-1α complexes.

System	Δ_b_ *G* (MMPBSA, kcal mol^−1^)		Δ(Δ_b_G)
	Normoxia	Hypoxia	
***Wild type***	−34 ± 12	−23 ± 12	**11**
**P138R**	−33 ± 08	−12 ± 10	**21**
**L163R**	−33 ± 09	−24 ± 10	**9**

MMPBSA, Molecular Mechanics Poisson-Boltzmann Surface Area MMPBSA.

Global structural fluctuations in protein backbones appear to be smaller under hypoxia (when HIF-1α Hyp564 is replaced by P564) with respect to normoxia (See **Figure S1** in the **Supplementary Material**). Differences in dynamic behavior among WT and P138R and L163R variants of pVHL are more pronounced under conditions representative of normoxia and accompanied by side-chain shifts in residues relevant for the pathophysiological functions of pVHL.

#### 3.6.1 Structure and Dynamics of VBC: HIF Involving Wild Type and P138R/L163R Variants

No major changes are detected in the tertiary and secondary structure of the pVHL: HIF complexes after introduction of variants P138R and L163R. Introducing variants affects specific interactions at the level of amino acid side chains directly in their local environment, and for L163R, it is propagated far away into the pVHL: HIF-1α interface. P138R introduces changes in a loop composed of residues 136–151.

#### 3.6.2 Flexibility of the Components of the Multiproteic Complex–Root-Mean-Square Fluctuation (RMSF)

pVHL backbone flexibility and VCB interunit adaptation in the VBC complex are essential features toward successfully recruiting Cullin 2 (Cul2) E3 ubiquitin ligase and HIF-1α (55). Under high oxygen conditions, P138R variant significantly increases pVHL backbone flexibility in the region around P138 substitution comprising residues 136–151 (**Figure S1**, left bottom). More precisely, while lining the floor of the β-domain in native pVHL, this flexibilized region constitutes a hydrophobic patch from where P138 establishes direct hydrogen-bonding interactions with H115 (one of the residues clamping Hyp564 from HIF-1α at the B-interface of pVHL) and Y112. In the P138R variant, the more extended and charged Arg138 lies at the bottom of the β-domain but displaced outward from the hydrophobic *core* and oriented toward helix H4. On the opposite direction, both variants slightly reduce the flexibility of the protein in the region 86–96, also in the β-domain of pVHL, as a part of the HIF-1α binding surface [primary binding site S1, quite shallow, rigid (13, 56)] including some of the well-conserved residues lining the Hyp564 binding cavity. No significant alterations are introduced by the L163R variant located in the α-domain of pVHL at the hydrophobic surface patch defining the interface with EloC where L163 establishes hydrophobic interactions with pVHL residues K159, L188, and a leucine from EloC. No significant alterations in flexibility are observed under conditions representative of hypoxia (**Figure S1**, panel C) other than a small reduction in the native protein around 86–96. Introducing variants in pVHL does not affect in a significant way HIF-1α flexibility (**Figure S1**, left bottom), which remains bound to VBC in all the cases with similar strengths under normoxia (**Table 2**). In the case of P138R, a small increase in flexibility is noticed under hypoxia in the region after Pro564, partially comprising the primary (S1) and secondary (S2) HIF-binding sites to pVHL. Introduction of variants in pVHL also reduces EloB flexibility in the region comprising residues 77–90. Whereas L163R does not alter EloC flexibility with respect to VBC formed with native pVHL, P138R induces a reduction mainly in the region defined by residues 83–93.

### 3.7 Changes Toward Molecular Interactions After Introducing Variants in pVHL

#### 3.7.1 Electrostatic Reorganization Influencing Molecular Recognition Properties

**Figure 6:**
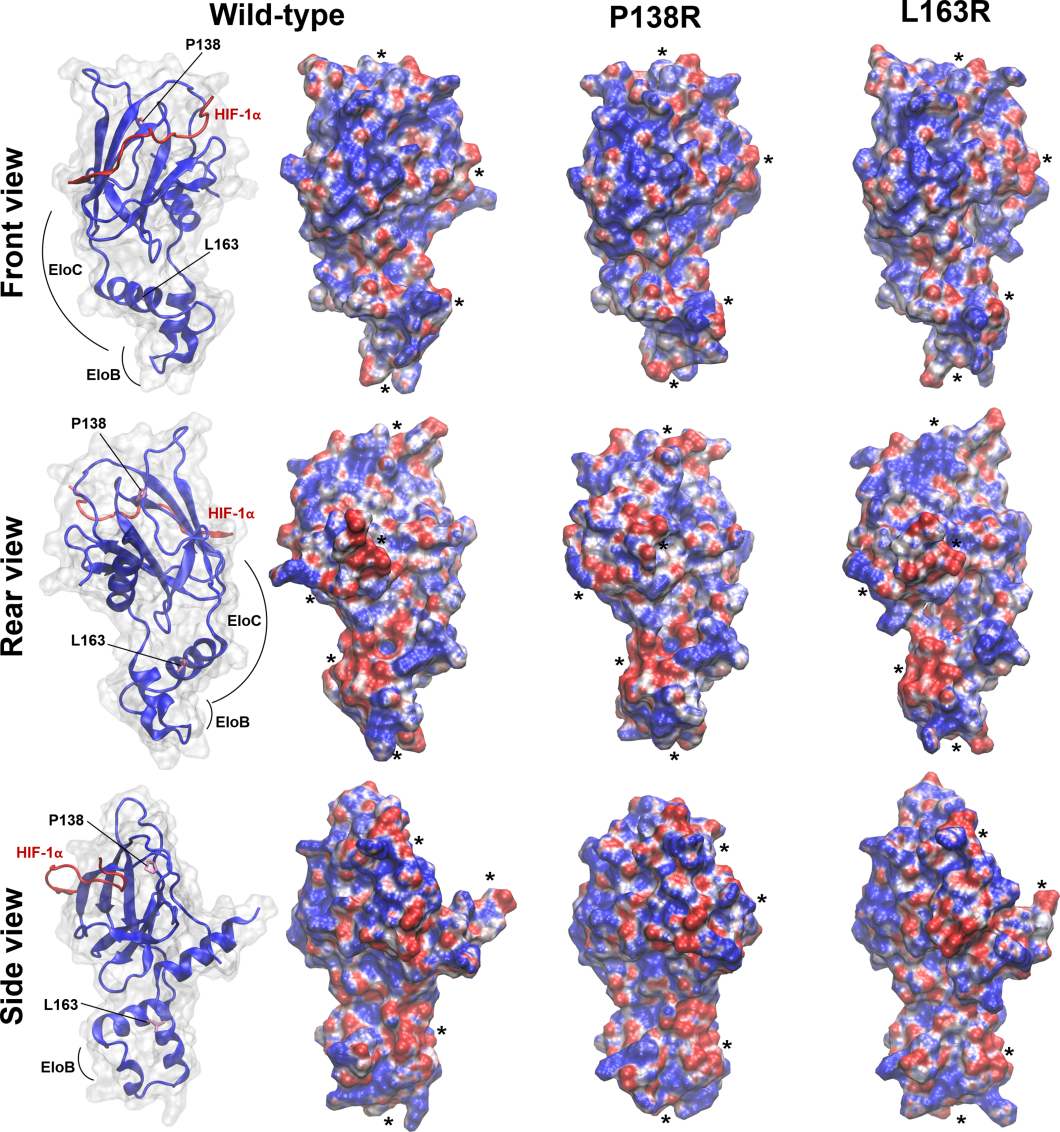
*In silico* studies showed both reorganization in shape and/or surface electrostatic potential in pVHL variants. Molecular electrostatic potential (MEP) is mapped on the Connolly surface as calculated for WT and P138R or L163R pVHL variants. Representative structures were extracted from the most populated cluster from each MD simulation. Units of potential range from -7 to 7 kT/e (red, negative values; blue, positive values). Relevant modifications in shape and/or surface MEP between WT and mutants are evidenced by placing black asterisks nearby. The interaction domains of pVHL with HIF and EloC/EloB are shown in the left for each of the three views displayed.

As shown in **Figure 6**, front-view representations, HIF-1α binding site in the native VBC complex has two regions of clearly defined positive and negative electrostatic potential that may be guiding HIF-1α recognition and proper positioning. Introduction of both variants in pVHL induces charge redistribution reflected in the molecular electrostatic potential (MEP) and changes in the surface molecular shape, with an influence in molecular recognition.

#### 3.7.2 Exposition to Solvent (SASA) of Relevant pVHL Lys Residues: K159, K171, and K196

We calculated the solvent-accessible surface area (SASA) for lysine residues 159, 171, and 196 (see **Figures 5B, C** for their location and orientation in each variant), which are targets for posttranslational modifications. **Figure S2** and **Table S1** in the **Supplementary Material** show the results for each of these. K159 is the most buried of the three Lys identified as relevant in the interaction with NEDD8. L163R variant further reduces solvent exposure of K159 in several frames of simulation, and this residue is reoriented. K171 is the most exposed of the three Lys inspected, and none of the variants affected its exposition. K196 is less exposed to solvent for the case of the L163R variant.

## 4 Discussion

In this study, we aimed to describe two novel variants of the VHL protein: P138R and L163R, which have been found in families with VHL disease and have not been functionally characterized before.

Firstly, by Western blot, we observed lower protein levels of the variants when compared to WT pVHL and showed that they have significantly lower half-lives compared to WT pVHL. Other groups have reported similar results for other pVHL variants such as S65W (57), N78S (57), Y98H (57, 58), W117A (26), P138L (59), V155A (60), L158P (57), L158Q (60), Q164R (60), R167Q (57, 61), R167W (58), L188Q (57), and L188V (60). There are striking differences among other authors’ results regarding the absolute value of WT pVHL and variant half-lives, even if we only consider those that use the same cycloheximide concentration (50 µg/ml). To compare our results with previous studies, we calculated the ratio between WT pVHL and our variants’ half-lives, resulting in 2.8 (P138R) and 3.4 (L163R) approximately. Lanikova et al. (59) have described P138L variant, obtaining different absolute values for the half-lives, but a similar ratio to the one reported here for P138R. If we compare mutations near L163R, Park et al. (58) have shown that Q164R’s half-life was reduced ≈3-fold compared to WT, while V155A and L158Q ≈5.5–6-fold. Ding et al. (61) showed a ≈3-fold reduction of R167Q’s half-life. When regarding absolute half-life values, Bangiyeva et al. (57) showed that after 2 h of cycloheximide treatment, levels of L158P and R167Q diminished drastically, becoming very low or undetectable by Western blot, resembling our results.

On the other hand, when cell lines were treated with the proteasome inhibitor MG132, we observed accumulation of WT pVHL, P138R, and L163R. Both variants increased their levels in a higher proportion than WT pVHL. Taken together, the above data suggest that the lower protein levels observed for VHL-P138R-Venus and VHL-L163R-Venus are due to proteasomal degradation.

The most studied mechanism for pVHL proteasome-mediated degradation is UCP-mediated polyubiquitination. Other authors have shown that UCP mediates the degradation of V155A, L158Q, and Q164R variants (60). P138R and L163R variants do not involve the substitution of lysine residues (subject to ubiquitination) directly, but they could alter their surroundings, favoring their exposure and thus their ubiquitination. Particularly for L163R variant, lysine 196 appears to be less exposed to the solvent, a result that would not favor polyubiquitination of this residue. Given that the region of interaction of pVHL with UCP has not been determined yet, one could speculate that this region might vary its conformation as a result of changes introduced in the pVHL protein. Therefore, an increase in the affinity of UCP for pVHL variants might explain their increased degradation compared to WT pVHL.

We showed that both pVHL variants maintain their ability to form a VBC complex, although it is apparently formed at a lower rate: P138R appears to bind less Elongin B and C, while L163R appears to bind Elongin C appropriately but less Elongin B. These results are in agreement with other groups’ findings, since the majority of inherited *VHL* mutations are defective in Elongin B and C binding (62–65). Other groups have shown that variants close to P138R and L163R such as D121G (66), Q145H (67), F148A (61), V155A (60), Q164R (60), and R167Q (61, 66, 68) form less VBC complex compared to WT pVHL, while L158P (69) and C162F (63, 70) are unable to form this complex and therefore do not have the capacity to downregulate HIF-α subunits (69, 71, 72).

On the other hand, VBC complex formation itself does not ensure its functionality, as it must recognize HIF-α subunits in order to target them for proteasomal degradation. Ding et al. (61) have shown that W117A and F148A mutations form less VBC complex and also lose their ability to interact with HIF-2α. We interrogated the capacity of the P138R and L163R pVHL variants to form a functional VBC complex and therefore accomplish the interaction and proteasome-mediated degradation of HIF-2α. By Western blot, intermediate levels of HIF-2α were observed by the cell lines expressing P138R and L163R; therefore, we decided to evaluate the consequence of these intermediate levels by evaluating the expression (mRNA) of two target genes: *VEGF-A* and *GLUT1*. We showed that under normoxic conditions, these genes exhibit the same regulation in cell lines expressing either the variants or WT pVHL. Nevertheless, after 24 h of pseudohypoxia, significant, though subtle, differences were observed between the cell lines expressing the variants compared to WT pVHL. As a consequence, variants’ VBC complexes could not appropriately regulate HIF-2α levels under these experimental conditions. This result suggests that the novel pVHL variants might have a different behavior compared to WT pVHL under more physiologically challenging conditions. The results obtained *in silico* suggest that VBC-HIF-1α complexes formed by the variants are thermodynamically favorable because of their negative ΔG.

In summary, our results indicate that although the protein levels for P138R and L163R pVHL variants are lower compared to WT pVHL, these interact forming a functional VBC complex capable of targeting HIF-2α for proteasome-mediated degradation.

As mentioned before, numerous pVHL HIF-independent mechanisms account for pVHL as a tumor suppressor (18, 19, 73). We decided to explore the relationship of these variants with fibronectin regulation, since it has been explored since 1998 and is the most described HIF-independent function to date (19). Other authors have shown that cell lines with pVHL mutant expression result in a defective fibronectin matrix deposition (19, 71, 74).

Our results indicate that although the novel variants exhibit a different regulation of fibronectin mRNA levels, they both fail in assembling a proper extracellular fibronectin matrix. For the L163R variant, less exposure to solvent of lysine 196 could explain a lower NEDDylation level and therefore the defective interaction with fibronectin, since NEDDylation has been described as a necessary switch for fibronectin interaction (75). These findings are speculative at this point and need to be tested *in vitro* in future studies.

The 786-0 cell line develops tumors when injected into nude mice, while clones of this cell line expressing WT-pVHL do not, or in some cases, they do but in a much smaller proportion of the injected mice compared to 786-0. Our xenograft experiments revealed that P138R-pVHL and L163R-pVHL failed to suppress tumor growth, obtaining 11 tumors out of 20 sites injected with each variant (55% incidence), a similar proportion to the one obtained by parental 786-0 cell line that does not express pVHL (40% incidence). These results confirm the pathogenic role for P138R and L163R pVHL variants, since they are unable to suppress tumor growth such as WT pVHL does. A study conducted by Ding et al. (61) revealed that the amount of a missense-mutated VHL protein (R167Q) could impact its function suppressing tumorigenesis when proteasome is inhibited, and this protein is therefore accumulated. Using the same approach and experimental tools, our pVHL variants were not able to compensate their functional deficiencies and demonstrated tumorigenic capacity, suggesting that there are a variety of mechanisms driving tumor formation. Our work reinforces the importance of studying specific variants to identify their biological impact. This work sets the stage for mechanistic studies exploring the altered mechanisms that explain pathogenesis and could lead to more targeted therapies for specific mutations.

Overall, our results show that P138R and L163R pVHL variants can be classified as pathogenic, since they failed to suppress tumor development in nude mice. Future studies are suggested for the elucidation of the mechanisms underlying their pathogenicity. In the current omics era, our study sets the basis for future proteomic and genomic approaches to compare cell lines expressing these variants with the WT protein to fully understand this missense variants’ global effects.

## Data Availability Statement

The raw data supporting the conclusions of this article will be made available by the authors without undue reservation.

## Ethics Statement

The animal study was reviewed and approved by Comité de Etica, Hospital de Niños Dr. R. Gutiérrez, Buenos Aires, Argentina.

## Author Contributions

PP conceived, designed, and directed the experimental research. CM, XL, and EJ designed the experiments. CM and MCF planned and carried out the experiments. AM collected data. AV, GS, and MB performed genetic and clinical characterization of VHL patients. ELC designed and directed the computational component of this work, and JB carried out all the molecular dynamics simulations. CM and MCF took the lead on writing the article under the supervision of PP and ELC (who wrote the *in silico* sections; contact laurac@fcien.edu.uy for direct inquiries). All authors provided critical feedback and helped shape the research, analysis, and article. All authors contributed to the article and approved the submitted version.

## Funding

This work was supported by Instituto Nacional del Cáncer, Ministerio de Salud, Argentina (Grant 2014-2016, awarded to PP) and Consejo Nacional de Investigaciones Científicas y Técnicas, CONICET, Argentina (PIP#0100214, 2013-2015, awarded to PP).

## Conflict of Interest

The authors declare that the research was conducted in the absence of any commercial or financial relationships that could be construed as a potential conflict of interest.

## Publisher’s Note

All claims expressed in this article are solely those of the authors and do not necessarily represent those of their affiliated organizations, or those of the publisher, the editors and the reviewers. Any product that may be evaluated in this article, or claim that may be made by its manufacturer, is not guaranteed or endorsed by the publisher.
