# Supplementary material for: VHL-P138R and VHL-L163R Novel Variants: Mechanisms of VHL Pathogenicity Involving HIF-Dependent and HIF-Independent Actions
Source: Front Endocrinol (Lausanne). 2022 Mar 21;13:854365. doi: 10.3389/fendo.2022.854365 (PMC8978939; doi:10.3389/fendo.2022.854365)
Supplement: Supplementary file 1 [file DataSheet_1.pdf]

Supplementary Material

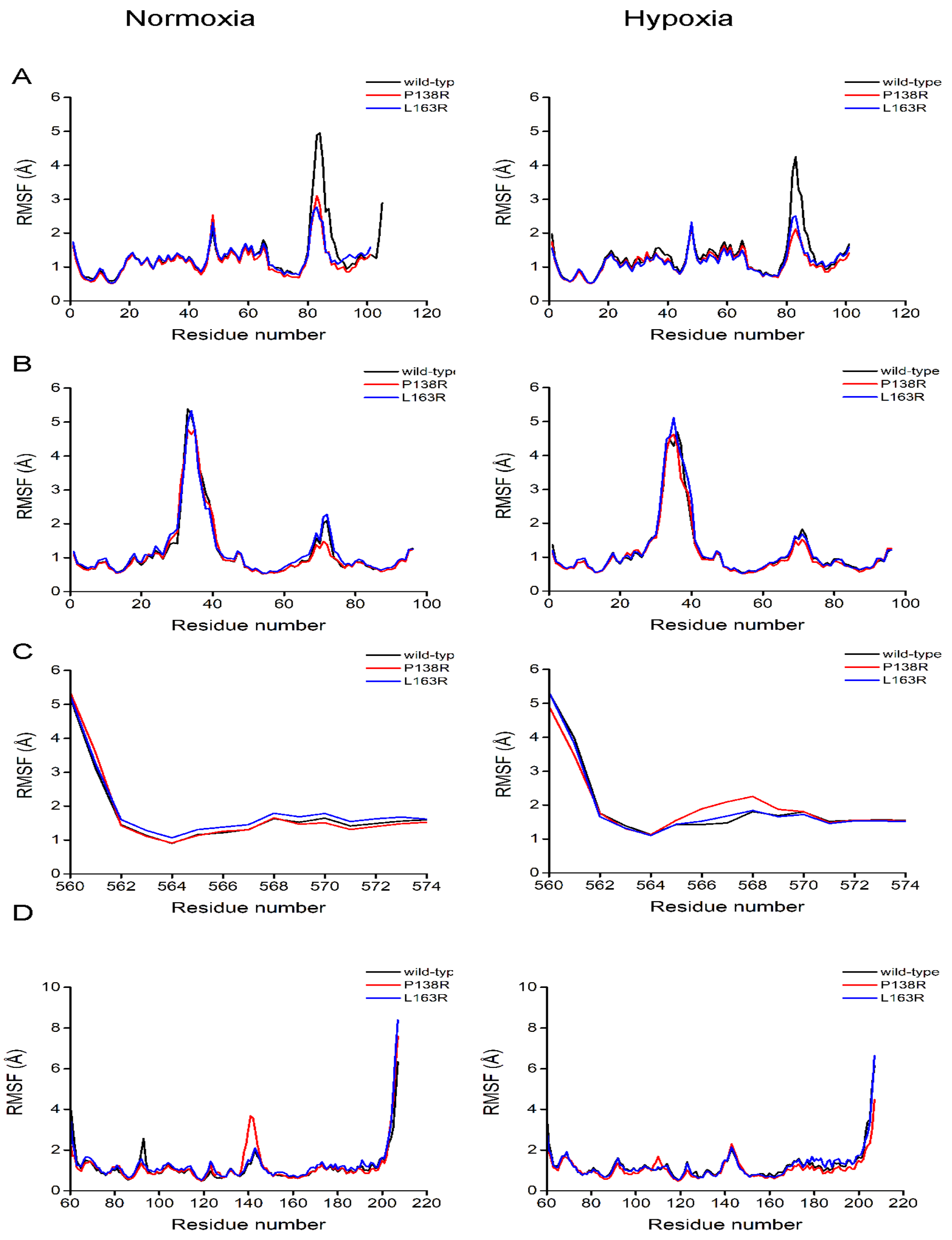

Figure S1. Changes in flexibility reported as Cα Root-Mean-Square Fluctuations (RMSFs) per residue extracted from MD trajectories obtained for each component of the VBC:HIF-1α complexes involving wild-type (black plots), P138R (red plots) or L163R (blue plots) under Normoxia or Hypoxia conditions (left and right plots for each component, respectively). A, B, C and D, correspond to EloB, EloC, HIF-1α and pVHL components.

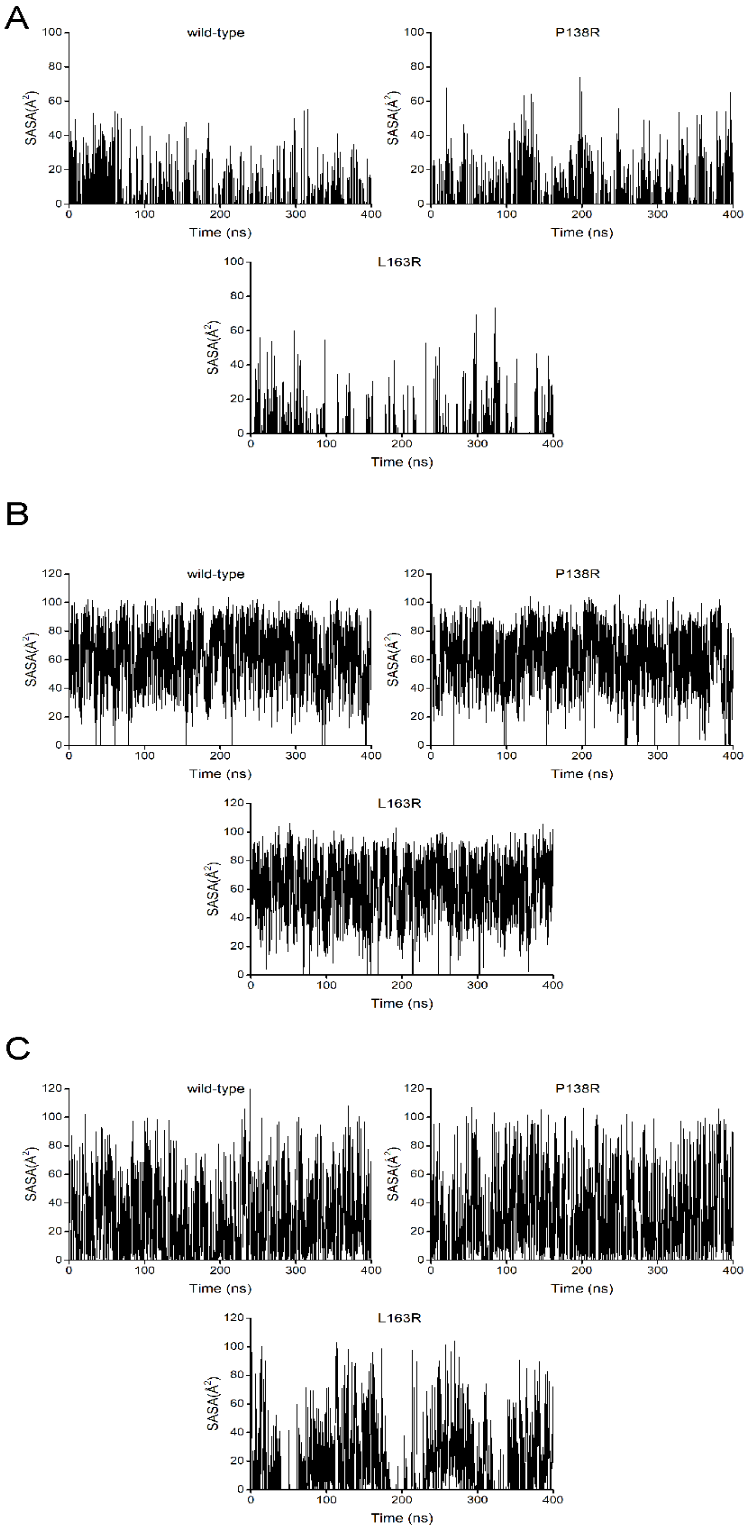

Figure S2. SASA (in Å^2) temporal evolution along the 400 MD trajectories for the side-chain of residues K151 (A), K179 (B) and K196 (C) in WT and P138R and L163R variants of pVHL under normoxia conditions.

Table S1. SASA values in Å^2 averaged along the MD simulations (with standard deviation) for lysines target for post-translational modifications in pVHL under normoxia

pVHL Variant	Averaged SASA (Å^2)		
	Lys159	Lys171	Lys196
**WT**	2 ± 7	63 ± 19	27 ± 24
**P138R**	4 ± 9	60 ± 19	31 ± 25
**L163R**	2 ± 6	61 ± 19	18 ± 22
